# Supplementary figures and images for: Exploring the effect of the triglyceride-glucose index on bone metabolism in prepubertal children, a retrospective study: insights from traditional methods and machine-learning-based bone remodeling prediction
Source: PeerJ. 2025 May 20;13:e19483. doi: 10.7717/peerj.19483 (PMC12101447; doi:10.7717/peerj.19483)

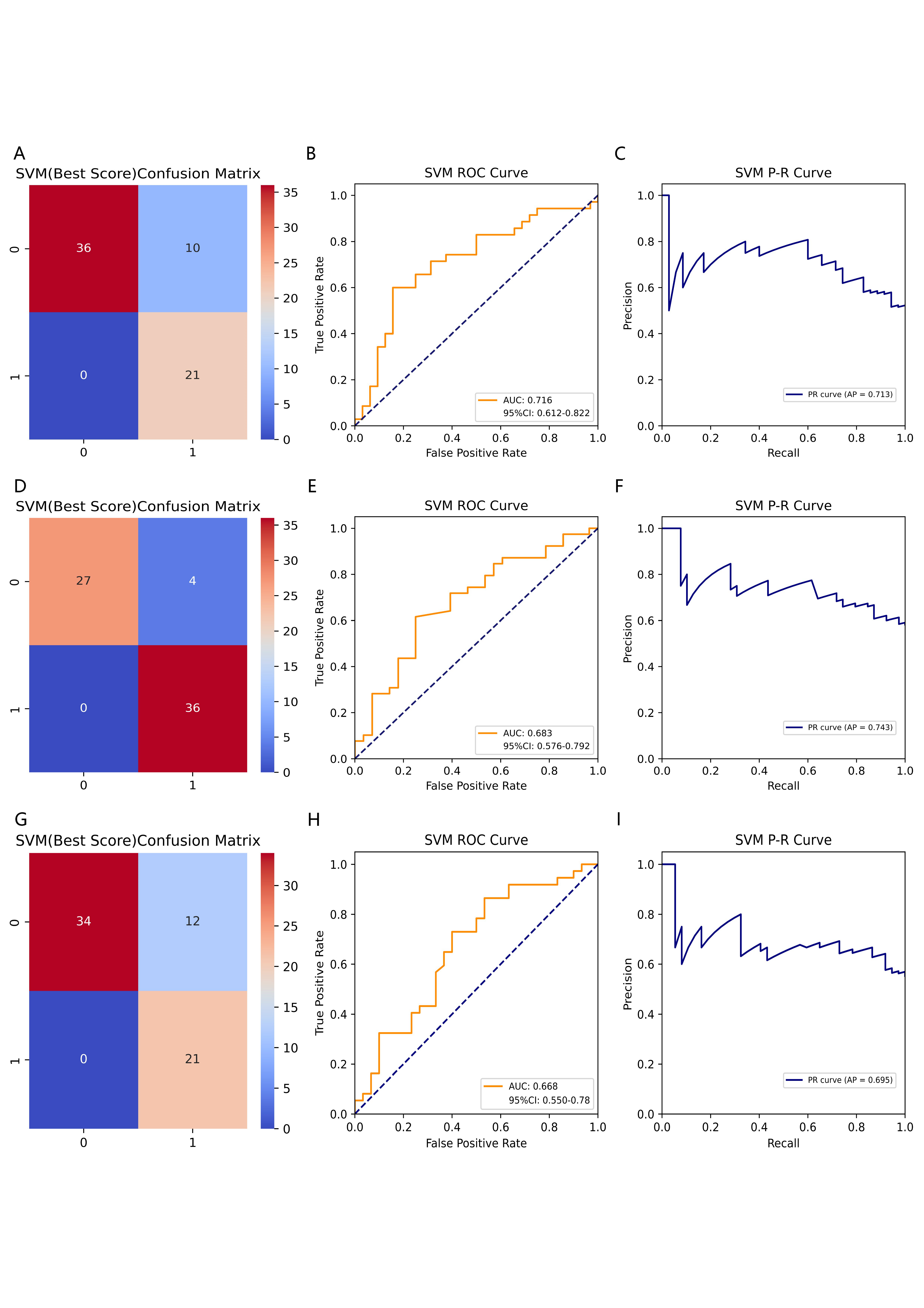

Supplement: Supplemental Information 5 — (A), (D), and (G) Denote the confusion matrices of SVM models predicting β-CTx, T-P1NP, and N-MID, respectively. (B), (E), and (H) Denote the ROC curves of SVM models predicting β-CTx, T-P1NP, and N-MID, respectively. (C), (F), and (I) Denote the P-R curves of SVM models predicting β-CTx, T-P1NP, and N-MID, respectively. [file peerj-13-19483-s005.png]

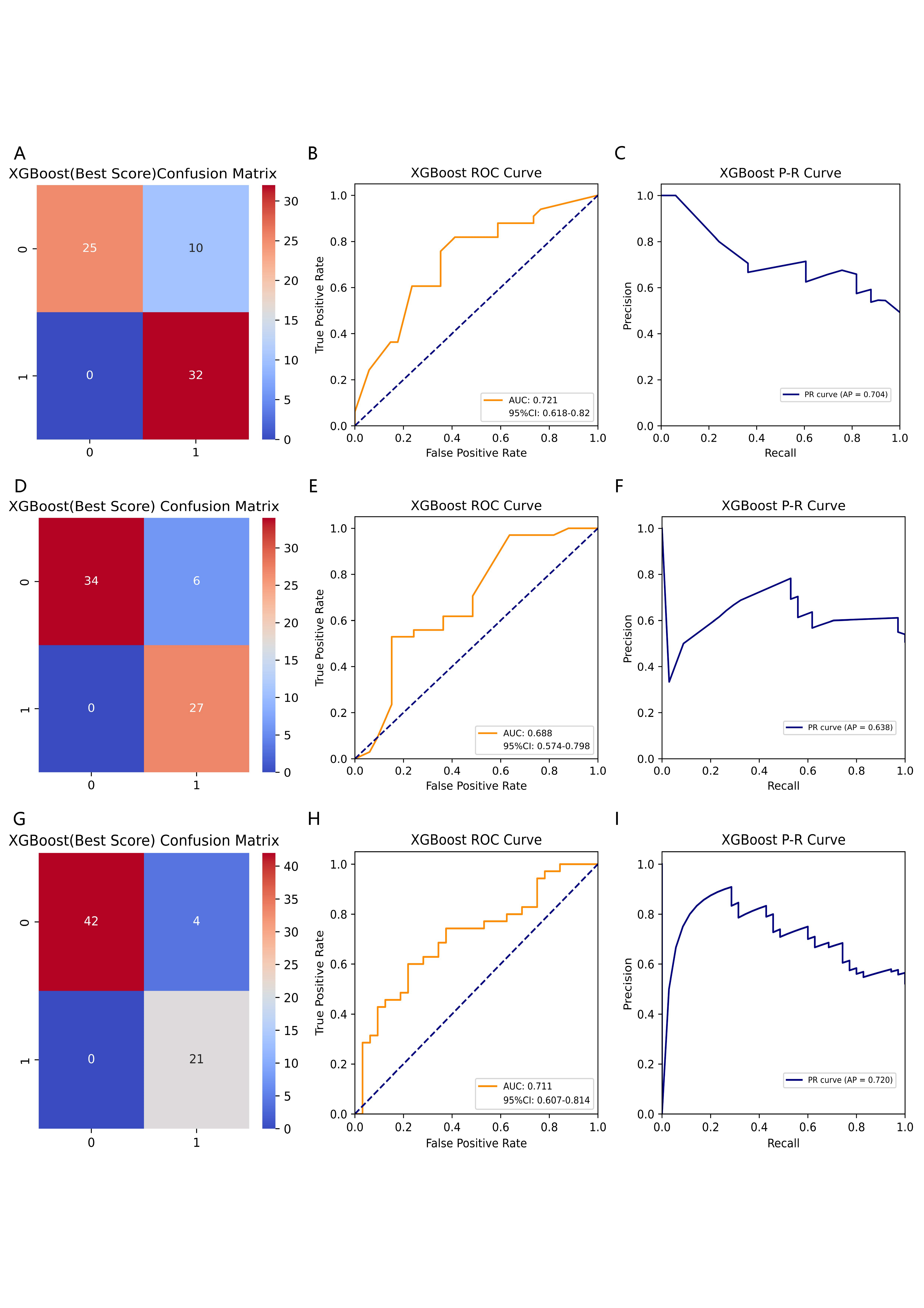

Supplement: Supplemental Information 6 — (A), (D), and (G) Denote the confusion matrices of XGBoost models predicting β-CTx, T-P1NP, and N-MID, respectively. (B), (E), and (H) Denote the ROC curves of XGBoost models predicting β-CTx, T-P1NP, and N-MID, respectively. (C), (F), and (I) Denote the P-R curves of XGBoost models predicting β-CTx, T-P1NP, and N-MID, respectively. [file peerj-13-19483-s006.png]

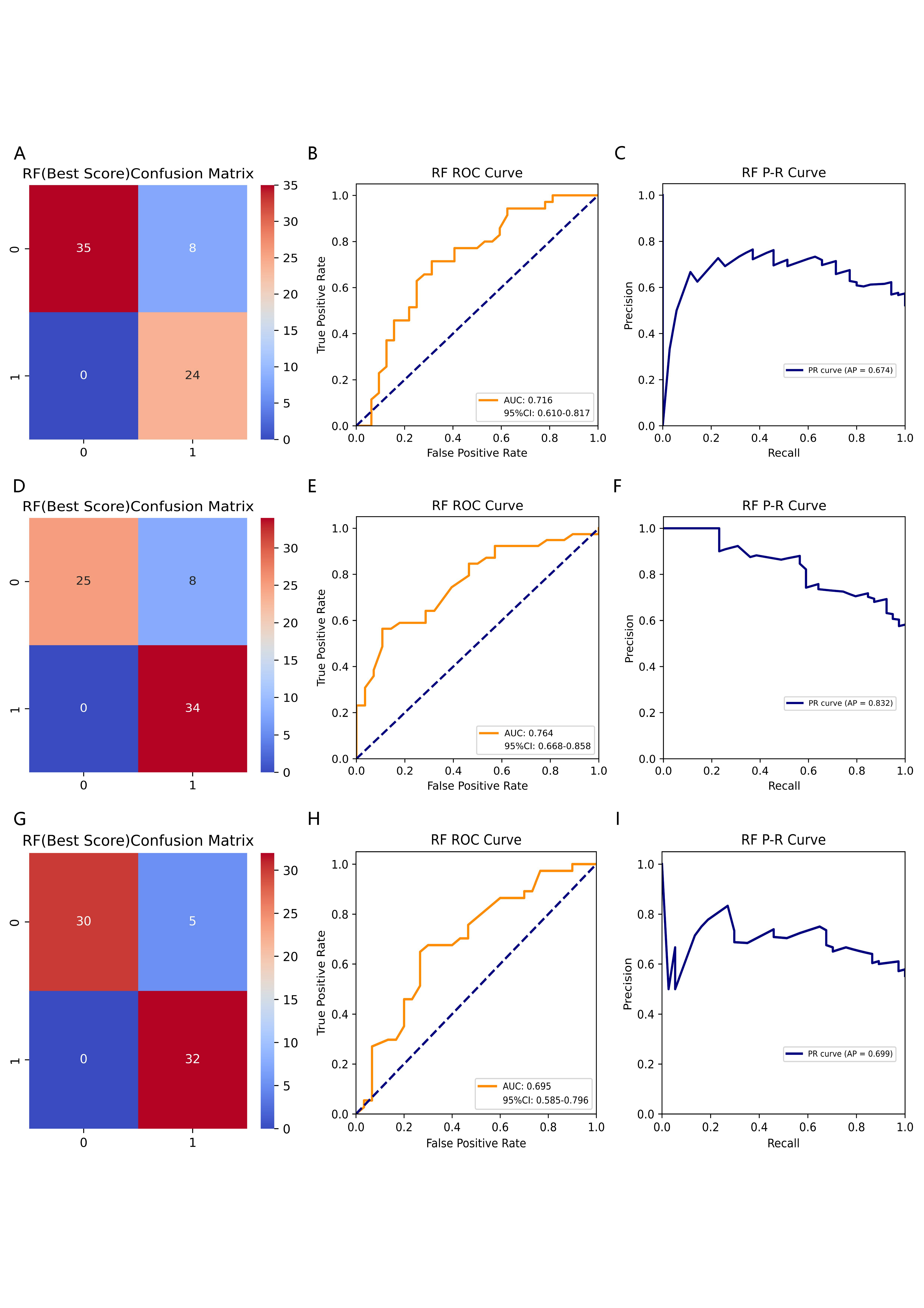

Supplement: Supplemental Information 7 — (A), (D), and (G) Denote the confusion matrices of RF models predicting β-CTx, T-P1NP, and N-MID, respectively. (B), (E), and (H) Denote the ROC curves of RF models predicting β-CTx, T-P1NP, and N-MID, respectively. (C), (F), and (I) Denote the P-R curves of RF models predicting β-CTx, T-P1NP, and N-MID, respectively. [file peerj-13-19483-s007.png]

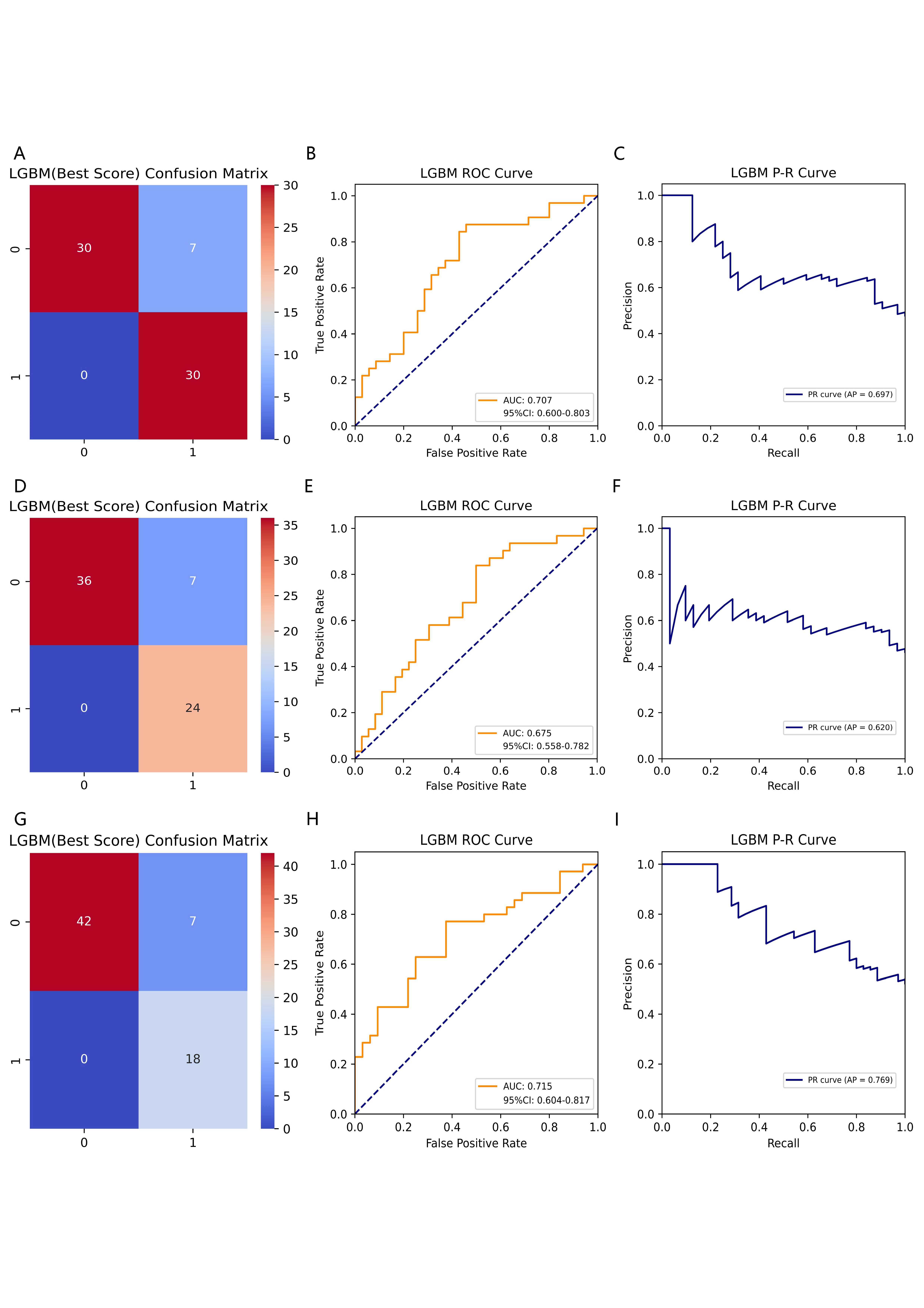

Supplement: Supplemental Information 8 — (A), (D), and (G) Denote the confusion matrices of LGBM models predicting β-CTx, T-P1NP, and N-MID, respectively. (B), (E), and (H) Denote the ROC curves of LGBM models predicting β-CTx, T-P1NP, and N-MID, respectively. (C), (F), and (I) Denote the P-R curves of LGBM models predicting β-CTx, T-P1NP, and N-MID, respectively. [file peerj-13-19483-s008.png]
