# Supplementary material for: Exploring the effect of the triglyceride-glucose index on bone metabolism in prepubertal children, a retrospective study: insights from traditional methods and machine-learning-based bone remodeling prediction
Source: PeerJ. 2025 May 20;13:e19483. doi: 10.7717/peerj.19483 (PMC12101447; doi:10.7717/peerj.19483)
Supplement: Supplemental Information 9 [file peerj-13-19483-s009.docx]

The initial variables include gender, age, weight, height, BMI, waist circumference, total BMD, TBLH BMD, TBLH BMD Z -score, lumbar spine BMD, total fat percentage, SII, HS- CRP, triglyceride, HDL, LDL, TC, FBG, insulin, TyG-BMI index, TyG index, HOMA-IR, HbA1c, spexin, FGF23, 1,25(OH)_2_D.
